# 3D Simultaneous Post-Contrast T1/T2 Mapping and Synthetic Multi-Contrast Late Gadolinium Enhancement at 0.55T: Validation in Porcine Myocardial Infarction Model

**DOI:** 10.1097/RLI.0000000000001277

**Published:** 2026-10-01

**Authors:** Dongyue Si, Juliet Varghese, Simon J. Littlewood, Katherine Binzel, Salman Pervaiz, Muhamad Mergaye, Mahmood Khan, Yuchi Han, Karl P. Kunze, Michael G. Crabb, Orlando P. Simonetti, Claudia Prieto, René M. Botnar

**Affiliations:** School of Biomedical Engineering and Imaging Sciences King’s College London, London, UK (D.S., S.J.L., K.P.K., M.G.C., C.P., R.M. B.); Department of Biomedical Engineering (J.V.); Dorothy M. Davis Heart and Lung Research Institute (K.B., S.P., M.M., M.K.); Department of Emergency Medicine (M.K., O.P.S.); Division of Cardiovascular Medicine, Department of Internal Medicine, The Ohio State University, Columbus, OH (Y.H., O.P.S.); MR Research Collaborations, Siemens Healthcare Limited, Camberley, UK (K.P.K.); Department of Radiology, The Ohio State University, Columbus, OH (O.P.S.); School of Engineering, Pontificia Universidad Católica de Chile (C.P., R.M.B.); Millennium Institute for Intelligent Healthcare Engineering (C.P., R.M. B.); Institute for Biological and Medical Engineering, Pontificia Universidad Católica de Chile, Santiago, Chile (R.M.B.); and Institute for Advanced Study, Technical University of Munich, Garching, Germany (R.M.B.).

**Keywords:** LGE, T1 mapping, T2 mapping, contrast enhancement, multi-contrast

## Abstract

**Objectives:**

To propose and validate a simplified method for 3D simultaneous post-contrast parametric mapping and synthetic late gadolinium enhancement (LGE) imaging at 0.55T for comprehensive whole-heart myocardial tissue characterization.

**Materials and Methods:**

A 3D joint T1/T2 mapping research sequence is adopted from a previous study. Three interleaved volumes with inversion recovery (IR) preparation, no magnetization preparation, and T2 preparation were acquired with image navigators to enable 100% respiratory scan efficiency. Intrinsically co-registered 3D T1, T2, and proton density maps were calculated using a dictionary-matching method, and Bloch equation-based IR and T2 preparation-IR (T2IR) signal models were proposed to generate multi-contrast 3D synthetic LGE images. In vivo evaluation included 10 data sets from a porcine myocardial infarction model to validate the performance of the proposed 3D method in comparison with that of separately scanned 2D reference sequences including post-contrast T1 mapping, pre-contrast T2 mapping, and LGE.

**Results:**

For the 10 swine data sets, 2D and 3D T1/T2 maps had consistent findings regarding the changes in T1/T2 values of myocardial infarction, presenting significantly decreased post-contrast T1 (2D: 279 ± 48 vs. 472 ± 44 ms, *P* < 0.01; 3D: 355 ± 32 vs. 597 ± 48 ms, *P* < 0.01) and increased T2 (2D: 102.4 ± 11.5 vs. 66.4 ± 3.1 ms, *P* < 0.01; 3D: 71.0 ± 5.3 vs. 39.4 ± 4.5 ms, *P* < 0.01) in scar compared with remote myocardium. 3D multi-contrast LGE images were successfully generated without additional scan and provided excellent image contrasts. Compared with 2D LGE, 3D synthetic bright-blood IR-LGE had improved scar-to-myocardium contrast (*P* < 0.01) with comparable image contrasts of scar-to-blood (*P* = 0.08) and blood-to-myocardium (*P* = 0.71), synthetic gray-blood IR-LGE had improved scar-to-blood and scar-to-myocardium contrast (*P* < 0.01) with comparable blood-to-myocardium contrast (*P* = 0.06), whereas synthetic dark-blood T2IR-LGE demonstrated significant differences regarding all tissue contrasts (*P* < 0.01).

**Conclusions:**

The proposed method provided imaging findings consistent with 2D references and shows promise for comprehensive myocardial tissue characterization in a single simple scan.

Cardiovascular magnetic resonance (CMR) has the ability to acquire multi-parametric and multi-contrast images for the detection of different cardiac diseases.^1^ Gadolinium-based contrast-enhanced imaging has been an important part of clinical CMR for myocardial tissue characterization,^2^ with late gadolinium enhancement (LGE) being the gold standard for non-invasive detection of myocardial scar.^3,4^ Conventional LGE is performed with an inversion recovery (IR) preparation pulse to induce T1-weighted contrast,^5^ with the inversion recovery time (TI) usually set to null the signal of normal myocardium to achieve bright scar visualization and a bright-blood contrast.^6^ Bright-blood LGE has an excellent scar-to-myocardium contrast for the detection of focal scarring. However, it may exhibit suboptimal contrast between scar and the adjacent blood pool, which can reduce sensitivity for the detection of subendocardial or papillary muscle myocardial infarctions.^7^ To further improve the scar-to-blood contrast in LGE, various sequences have been proposed to suppress the blood signal.^8-11^ Some techniques use IR preparation with TI set to null the blood pool signal, which achieves gray-blood LGE after phase-sensitive reconstruction.^11,12^ Other sequences create dark-blood contrast by combining IR and additional preparation pulses, such as T2 preparation.^13,14^ The signal of blood and myocardium can be simultaneously nulled by optimizing both T1 and T2 contrast weightings in LGE.

Compared with various contrast weighted LGE imaging methods that provide excellent delineation of focal scar, emerging CMR parametric mapping techniques enable quantification of tissue related MR parameters such as T1 and T2, thereby offering superior performance for the detection of diffuse conditions such as myocardial fibrosis and edema.^4,15-17^ Therefore, simultaneous imaging of parametric mapping and LGE is preferred for comprehensive evaluation of both focal and diffuse disease processes. Currently, clinically available CMR imaging techniques can only acquire one parameter or contrast per scan, and most sequences remain limited to 2D breath-hold acquisitions with relatively low acquisition efficiency.^17,18^ Therefore, an efficient and simplified CMR technique that allows 3D simultaneous multi-parametric and multi-contrast imaging in a single simple scan is of high value in clinical practice.

Calculation of synthetic LGE images has been proposed in previous studies using post-contrast T1 map to simulate IR prepared T1-weighted images, which presented excellent agreement with conventionally acquired LGE.^19-21^ More recently, simultaneous multi-parametric mapping sequences were proposed for efficient imaging of 2 or more parameters such as T1 and T2.^22-24^ On the basis of joint T1/T2 mapping, an all-in-one approach was also proposed using T1 and T2 to calculate both bright-blood and dark-blood LGE images.^25^ However, 2D imaging with a relatively thick imaging slice and reduced spatial coverage limited the application for high-resolution whole-heart assessment.

Previous experiences with parametric mapping and synthetic LGE are mostly based on images acquired at common MR field strengths such as 1.5T or 3T.^25,26^ In recent years, there has been renewed interest in CMR imaging at low field given the intrinsic advantages of lower costs, larger bore size, lower specific absorption rates, and improved B_0_ and B_1_ field homogeneities.^27-30^ A highly efficient 3D free-breathing joint T1/T2 mapping sequence was proposed on a 0.55T commercial scanner,^31^ and was able to acquire whole-heart T1 and T2 maps with good image quality and 2 mm isotropic resolution in 7 minutes. However, the shorter T1 and longer T2 relaxation times at low-field MRI may have different imaging characteristics for multi-contrast LGE compared with common field strengths.^27^ In this study, we aim to validate the feasibility of 3D joint T1/T2 mapping for post-contrast imaging and synthetic multi-contrast LGE at 0.55T.

## MATERIALS AND METHODS

### Joint T1/T2 Mapping

The 3D joint T1/T2 mapping research sequence is adopted from a previous study.^31^ Three electrocardiogram-triggered water/fat volumes are acquired with Dixon gradient echo readout using IR preparation (TI = 150 ms), no magnetization preparation and T2 preparation (50 ms), respectively, in a repeated scheme of 3 interleaved heartbeats (Fig. 1A). 2D image navigators are acquired in each heartbeat to detect respiratory motion in left-right and superior-inferior directions, enabling 100% respiratory scan efficiency.^32^ A variable-density Cartesian trajectory with spiral-like profile order and golden-angle step is adopted with 4-fold undersampling.^33^ The 3 volumes are reconstructed using an inline nonrigid motion-corrected iterative SENSE reconstruction with vendor-implemented Dixon water/fat separation, followed by offline high-dimensional patch-based low-rank regularization denoising.^34,35^ Intrinsically co-registered 3D T1 and T2 maps are jointly calculated voxel-by-voxel from the 3 water volumes using a dictionary-matching method (Fig. 1B). A specific dictionary is generated for each subject with Bloch equation simulations to calculate the signal intensity of each volume considering the subject’s heart rate and acquisition parameters. Proton density is estimated with the scaling factor between the measured signal intensity of the 3 acquired volumes and the related signal intensity in the dictionary.^36,37^

### Synthetic Multi-Contrast LGE

Multi-contrast LGE images were generated voxel-by-voxel with proton density and T1 and T2 maps by signal simulation using the following IR and joint T2 preparation-IR (T2IR) signal model, respectively (Fig. 1B):

$$\text{IR signal model:}\;\;S_{\text{IR}-\text{LGE}}=\text{PD}(1-2e^{-\frac{\text{TI}}{T_{1}}})\;\;\text{T2IR signal model:}\;\;S_{\text{T2IR}-\text{LGE}}=\text{PD}[1-(1+e^{-\frac{\text{TE}}{T_{2}}})e^{-\frac{\text{TI}}{T_{1}}}]$$

Where S is the calculated signal intensity of synthetic IR-LGE and T2IR-LGE, respectively, PD is proton density, TI is the inversion recovery time, and TE is the T2 preparation time. In this study, IR-LGE images with bright-blood contrast were generated by selecting TI to null myocardium, that is, $S_{\text{IR}-\text{LGE},\text{myo}}={\text{PD}}_{\text{myo}}\left(1-2e^{-\frac{\text{TI}}{T_{l,\text{myo}}}}\right)=0$, deriving $\text{TI}=T_{l,\text{myo}}\times \text{ln}2$. Similarly, IR-LGE with gray-blood contrast was generated by selecting TI to null blood pool, that is, $S_{\text{IR}-\text{LGE},\text{blood}}={\text{PD}}_{\text{blood}}\left(1-2e^{-\frac{\text{TI}}{T_{l,\text{blood}}}}\right)=0$, deriving $\text{TI}=T_{l,\text{blood}}\times \text{ln}2$. T2IR-LGE images with dark-blood contrast were generated using TE and TI, nulling myocardium and left ventricle blood pool simultaneously, that is, $S_{\text{T2IR}-\text{LGE},\text{myo}}=S_{\text{T2IR}-\text{LGE},\text{blood}}=0$. As an analytical solution is very complicated and unnecessary, numerical simulations were performed to solve for TE and TI.^26,38^

### Study Design

Six swine were included in this study for CMR imaging at 1-week and 5-week post-myocardial infarction to validate the performance of the proposed method in comparison with 2D breath-held T1 and T2 mapping and LGE imaging. An ischemia-reperfusion model, involving a 90-minute occlusion followed by reperfusion of the left circumflex artery, was used to induce myocardial infarction. In each CMR session, 2D T2 mapping was acquired before contrast injection, whereas 2D LGE, 2D T1 mapping and 3D joint T1/T2 mapping were performed sequentially about 10 minutes after administration of a total dose of 0.2 mmol/kg of gadobutrol (GADAVIST, Bayer Healthcare, Leverkusen, Germany). The 3D joint T1/T2 mapping sequence used the same imaging parameters as shown in a previous study.^31^ 2D T1 and T2 mapping were performed with segmented modified Look-Locker inversion recovery and T2-prepared balanced steady-state free precession (bSSFP), respectively.^39,40^ Segmented acquisitions were utilized for the 2D T1 and T2 maps in this study to minimize cardiac motion artifacts arising from the higher heart rates in the swine and to achieve adequate spatial resolution for tissue characterization. The 2D maps were acquired in a slice each of 2, 3, and 4-chamber long-axis views, as well as in 3 short-axis slices covering the base, middle, and apical portion of the left ventricle. 2D bright-blood contrast LGE was acquired in a stack of 8 to 10 short-axis slices and 3 long-axis slices with a breath-held segmented phase-sensitive inversion recovery sequence. Imaging parameters for all sequences are summarized in Table S1, Supplemental Digital Content 1, http://links.lww.com/RLI/B97.

MRI experiments were performed with a 0.55T MR scanner (MAGNETOM Free.Max, Siemens Healthineers, Forchheim, Germany) using a 6-channel and 12-channel chest array receiver coils placed posterior and anterior to the animal, and an external electrocardiogram monitoring system (Expression MR400, Philips N.V, Amsterdam, The Netherlands). All animal studies were approved by the local Institutional Animal Care and Use Committee (2012A00000019). Animals were anesthetized with isoflurane and mechanically ventilated with 50% oxygen throughout the MRI study. Breath-holding required for 2D cardiac sequences was achieved by pausing the ventilator.

### Data Processing and Analysis

Image processing was implemented in MATLAB R2023a (The MathWorks, Natick, MA), and statistical analysis was performed using SPSS Statistics 29 (IBM, Armonk, NY). To prepare the data for analysis, the 3D joint T1/T2 maps were reformatted to multiple short-axis and long-axis slices matching the orientations of the conventional 2D LGE sequences. Approximately 30 short-axis slices with 2 mm isotropic resolution were generated for each data set. To estimate T1/T2 values of normal myocardium and/or blood pool for the parameter selection of synthetic LGE, a region of interest (ROI) of the related tissue was manually defined in a representative short-axis T1/T2 map, and the mean value within each ROI was calculated. Finally, synthetic bright-blood and gray-blood IR-LGE and dark-blood T2IR-LGE images associated with joint T1/T2 maps were generated using the IR and T2IR signal model described before.

Bull’s-eye plot analysis was performed with the 3D T1 and T2 maps and synthetic LGE images. The epicardial and endocardial contours of the left ventricle were manually segmented on all the short-axis images. As 3D T1/T2 maps and LGE images measured by the proposed sequence were intrinsically co-registered, the contours were segmented on T2 maps and then transferred onto the other corresponding images. High-resolution bull’s-eye plots were generated with 36 sectors per short-axis slice. For the T1/T2 map, the left ventricle was also divided into 16 segments according to the American Heart Association (AHA) 17-segment model,^41^ and the mean T1/T2 value within each segment was calculated. The apical segment was excluded to avoid partial volume effect.

To evaluate the performance of post-contrast T1 and T2 mapping, T1 and T2 values of myocardial infarction scar and remote myocardium were measured and compared for both 2D and 3D sequences. ROI of the related tissue was manually defined in a representative short-axis slice to estimate the mean T1/T2 value. A paired 2-tailed Student *t* test (α = 0.05) was used to analyse the statistical differences between the T1/T2 values of different tissues measured by each sequence, respectively. Bland-Altman analysis was also performed to evaluate the differences between 2D and 3D results.

To evaluate the performance of synthetic LGE imaging, scar detection analysis was performed comparing the results of 3D synthetic multi-contrast LGE and the reference 2D LGE. The assessment was performed by an experienced reader (7 y of CMR experience). Scar detection analysis was carried out on each data set using the 17-segment AHA model. Specifically, all short-axis slices covering the left ventricle were divided into 17 AHA segments, and each segment was visually inspected to identify scar.^42^

Furthermore, the contrast between scar, blood, and remote myocardium was calculated for 2D LGE and 3D synthetic bright-blood IR-LGE and dark-blood T2IR-LGE. A representative short-axis slice that showed scar was selected for each data, then, ROIs of scar, blood, and myocardium were manually drawn to measure the average signal intensity (S) of each tissue, respectively. Given 2 tissues, A and B, selected from scar, blood, and myocardium, the A-to-B relative contrast is defined as:

$${\text{Contrast}}_{A-B}=\frac{S_{A}-S_{B}}{\sqrt{S_{\text{Scar}}^{2}+S_{\text{Blood}}^{2}+S_{\text{Myo}}^{2}}}$$

where $S_{A}$ is the signal intensity of tissue A and $S_{B}$ is the signal intensity of tissue B. The contrast of scar-to-blood, scar-to-myocardium and blood-to-myocardium were calculated, and the statistical differences between tissue contrasts measured with 2D and 3D sequences were analyzed using a paired 2-tailed Student *t* test (α = 0.05).

## RESULTS

All 6 swine (~2 to 3 mo old at baseline and weighing 30 to 40 kg over the study duration) successfully underwent the CMR scan at 1-week post-myocardial infarction. One swine did not survive until the 5-week scan, and another had failed contrast injection during the 5-week scan, leading to successfully acquired 5-week data of 4 swine. Overall, 10 swine CMR data sets were included for analysis. The proposed 3D joint T1/T2 mapping sequence was acquired within a scan time of 5.0 ± 1.0 minutes with an average heart rate of 102 ± 19 bpm. The scan time and imaging time after contrast administration of different sequences are summarized in Table S2, Supplemental Digital Content 1, http://links.lww.com/RLI/B97. All reference 2D LGE images detected myocardial infarction scar at the inferior-lateral segment, which was associated with the territory of the left circumflex artery that underwent occlusion and reperfusion.

Representative post-contrast T1/T2 maps and synthetic multi-contrast LGE images of a swine produced with the proposed method are shown in Figure 2. According to visual comparison (Fig. 2A), synthetic LGE calculated with proton density map demonstrated a cleaner background air region than that without proton density correction, and provided results comparable to the reference 2D LGE image. Using different signal models and varying parameters, multiple LGE images with different contrasts were calculated without additional acquisitions (Fig. 2B). The IR signal model enabled the calculation of synthetic LGE images nulling either blood or myocardium by changing TI. In contrast, the T2IR signal model allowed the calculation of synthetic dark-blood LGE images nulling both blood and myocardium by selecting appropriate TE and TI.

Representative images of another swine acquired with the proposed method in comparison with reference 2D LGE are shown in Figure 3. The proposed sequence provided intrinsically co-registered post-contrast T1/T2 maps and synthetic bright-blood, gray-blood, and dark-blood LGE images of the whole heart with good image qualities. The scar area demonstrated decreased T1 and increased T2 in the post-contrast T1/T2 maps. Visual inspection showed that the synthetic bright-blood IR-LGE had good image quality and tissue contrast for scar detection comparable with reference 2D LGE, although the in-plane resolution of 3D imaging is slightly lower than that for 2D LGE (2×2 vs. 2.1×1.5 mm^2^). The synthetic dark-blood T2IR-LGE does not have sufficient contrast for the depiction of cardiac structure as both blood and myocardium were suppressed; however, it demonstrated excellent contrast to identify scar. Although the synthetic gray-blood IR-LGE had improved tissue contrasts for the depiction of all tissues. The corresponding high-resolution bull’s-eye plots of this swine are shown in Figure 4. Post-contrast T1/T2 maps and synthetic LGE images showed consistent imaging findings in the segments with scar, demonstrating decreased T1 and increased T2 values and hyperintensity in the LGE images, respectively. High-resolution plots facilitated a more accurate depiction of the scar area across the whole heart.

Short-axis images acquired from 2 swine at 1-week post-myocardial infarction are shown in Figure 5. 3D post-contrast T1/T2 maps and LGE images generated with the proposed sequence had scar detection performance comparable to reference results acquired with 2D sequences. For the first swine, 2D LGE showed a hyperintense area with a hypointense core, indicating myocardial infarction with microvascular obstruction (MVO), which was not easily identified on 2D post-contrast T1 and pre-contrast T2 maps. As the MVO area is relatively small and only 3 short-axis slices are acquired with 2D T1 and T2 mapping, it may be possible that the related slice was missed by the 2D sequences. Both 3D synthetic bright-blood IR-LGE and dark-blood T2IR-LGE successfully visualized the MVO, although with a slightly different area size compared with 2D LGE. This is potentially due to the kinetics of contrast agent washout, as the 3D sequence was always acquired after the 2D sequence. The area of MVO was also visible in the 3D post-contrast T1 map with a relatively higher value than that of scar, whereas the post-contrast T2 map provided a relatively lower contrast for MVO detection.

Quantitative comparison of post-contrast T1 and T2 values for different tissues is shown in Figure 6 and summarized in Table 1. 2D and 3D T1/T2 maps had consistent findings for the detection of scar. Post-contrast T1 of scar was significantly lower than that of remote myocardium according to both 2D T1 (279 ± 48 vs. 472 ± 44 ms, *P* < 0.01) and 3D joint T1/T2 mapping (355 ± 32 vs. 597 ± 48 ms, *P* < 0.01). Although T2 of scar was significantly higher than that of remote myocardium according to both 2D pre-contrast T2 (102.4 ± 11.5 vs. 66.4 ± 3.1 ms, *P* < 0.01) and 3D post-contrast joint T1/T2 mapping (71.0 ± 5.3 vs. 39.4 ± 4.5 ms, *P* < 0.01). The absolute T1/T2 values measured by 2D and 3D joint T1/T2 mapping sequences were different (Figure S1, Supplemental Digital Content 1, http://links.lww.com/RLI/B97). Post-contrast 3D T1 was higher than 2D T1 with bias of 124.3 and 75.4 ms for remote myocardium and scar, respectively. Although post-contrast 3D T2 was lower than pre-contrast 2D T2 with bias of −27.1 and −31.4 ms for remote myocardium and scar, respectively.

Scar detection analysis with the 17-segment AHA model was performed for all 10 data sets. Excellent agreement in scar detection and scar location across the whole heart was obtained between the 2D and 3D multi-contrast LGE images with 100% consistent findings in all 170 segments. Regarding image contrast between different tissues (Fig. 7), 3D bright-blood IR-LGE and reference 2D LGE were not significantly different in the contrast of scar-to-blood (0.227 ± 0.304 vs. 0.096 ± 0.154, *P* = 0.08) and blood-to-myocardium (0.566 ± 0.192 vs. 0.582 ± 0.106, *P* = 0.71), whereas scar-to-myocardium contrast of 3D bright-blood IR-LGE was slightly higher than that of 2D LGE (0.793 ± 0.113 vs. 0.678 ± 0.081, *P* < 0.01). 3D gray-blood IR-LGE had significantly higher scar-to-blood (0.479 ± 0.397 vs. 0.096 ± 0.154, *P* < 0.01) and scar-to-myocardium (1.220 ± 0.195 vs. 0.678 ± 0.081, *P* < 0.01) contrast than reference 2D LGE, whereas the blood-to-myocardium contrast was comparable (0.741 ± 0.297 vs. 0.582 ± 0.106, *P* = 0.06). As expected, 3D dark-blood T2IR-LGE and reference 2D LGE demonstrated significant differences regarding scar-to-blood contrast (1.000 ± 0.003 vs. 0.096 ± 0.154, *P* < 0.01) and blood-to-myocardium contrast (−0.022 ± 0.023 vs. 0.582 ± 0.106, *P* < 0.01). Furthermore, dark-blood T2IR-LGE also had improved scar-to-myocardium contrast (0.978 ± 0.025 vs. 0.678 ± 0.081, *P* < 0.01).

## DISCUSSION

In this study, a simplified and highly efficient method for simultaneous post-contrast parametric mapping and synthetic LGE imaging in a single scan was proposed based on a 3D free-breathing joint T1/T2 mapping sequence developed and implemented at 0.55T. Intrinsically co-registered 3D T1, T2, and proton density maps were calculated using a dictionary-matching method, and Bloch equation-based IR and T2IR signal models were proposed to generate multi-contrast synthetic 3D LGE images using T1, T2, and proton density. In vivo validation experiments with a porcine myocardial infarction model showed that the proposed method can acquire 3D images with 2 mm isotropic resolution in about 5 minutes. 3D T1 and T2 maps showed consistent findings with the reference 2D results, demonstrating significantly reduced T1 and elevated T2 in scar regions. The 3D synthetic bright-blood IR-LGE provided image contrasts comparable with the reference 2D LGE, whereas synthetic gray-blood IR-LGE and dark-blood T2IR-LGE demonstrated improved contrast.

The accurate measurement of myocardial T1 and T2 maps is the basis of synthetic LGE imaging. The adopted 3D joint T1/T2 mapping sequence has been proposed and validated at 0.55T in a previous study including phantom, healthy volunteers and a patient, which demonstrated that the sequence has good accuracy and precision for both T1 and T2 measurements.^31^ Here, this sequence was adopted for post-contrast imaging of swine at 0.55T, which had 2 main differences compared with the previous study. Firstly, gadolinium-based contrast agent will significantly shorten tissue T1, which is beneficial for the sequence as the signal will recover faster after IR preparation, increasing the signal-to-noise ratio. Secondly, the swine presented a higher heart rate of 100 bpm compared with that of healthy volunteers. Nevertheless, phantom experiments in the previous study showed that T1 and T2 measured utilizing the proposed sequence were not sensitive to heart rate variation and maintained good accuracy and precision. In summary, the proposed 3D joint T1/T2 mapping is feasible for post-contrast swine imaging at 0.55T.

Comparing T1 and T2 values measured by 2D and 3D sequences in this study, there were some differences regarding the absolute T1 and T2 mean values for the different tissues (Figure S1, Supplemental Digital Content 1, http://links.lww.com/RLI/B97). Post-contrast T1 measured with 3D joint T1/T2 mapping was higher than that measured with 2D T1 mapping. As a prior study demonstrated comparable T1 measurement accuracy between 3D joint T1/T2 mapping and the 2D reference sequence,^31^ this bias in post-contrast T1 should mainly be due to the differences in imaging time after contrast administration. 3D joint T1/T2 mapping was performed after 2D T1 mapping in the study, thus the bias reflects the T1 increases with the washout of contrast agent over time. Post-contrast T2 measured with 3D joint T1/T2 mapping was lower than pre-contrast T2 measured with the T2-prepared 2D bSSFP mapping sequence. It is known that T2-prepared bSSFP tends to overestimate T2 especially for shorter T1 values due to the bSSFP influence,^43^ whereas 3D joint T1/T2 mapping has better accuracy.^31^ Furthermore, the contrast agent may also slightly shorten tissue T2,^44^ causing the difference between pre-contrast and post-contrast T2 measurements. However, T2 shortening is usually more obvious with higher gadolinium concentrations.^44^ Overall, according to preliminary results in this study, the bias in the mean T1/T2 value did not influence the diagnostic performance of each sequence to identify scar, that is, T1 and T2 measured by both 2D and 3D sequence showed significant differences between scar and remote myocardium. The promising results indicate that post-contrast 3D joint T1/T2 mapping could be used for the detection of scar. Given the bias in the absolute T1/T2 values measured by different sequences, sequence-specific reference values of normal myocardium should be measured before applying the sequence to clinical diagnosis. This should also include a reference extravascular volume (ECV) value that calculated from the pre-contrast and post-contrast T1, when applicable.

With accurate T1 and T2 maps, synthetic LGE images can be calculated without additional LGE-specific image acquisitions. Usually, a TI scout scan is performed before LGE imaging for manually TI selection, a task that relies on operator skills,^18^ and may require repeated scans if the LGE contrast is suboptimal. Although for synthetic LGE, the sequence parameters (TI and/or TE) can be appropriately selected based on the quantitative T1 and T2 values of the target tissues, rather than relying on manual adjustments. This simplifies the scan procedure and reduces the total planning and scanning time. In addition, retrospective adjustment of sequence parameters allows the generation of multi-contrast LGE images from a single acquisition, enhancing image interpretability and diagnostic assessment. Both IR and T2IR signal models were proposed to make full use of T1 and T2 maps and calculate bright-blood, gray-blood and dark-blood LGE images in this study. The synthetic bright-blood IR-LGE had image contrast comparable to the reference 2D LGE except that the scar-to-myocardium contrast was slightly improved because the signal of the myocardium was completely nulled using the optimal TI. However, bright-blood LGE had relatively lower scar-to-blood contrast (close to 0). Synthetic gray-blood IR-LGE improved both the scar-to-blood and scar-to-myocardium contrast while maintaining the blood-to-myocardium contrast, thus might be optimal for simultaneous depiction of all 3 tissues. For the synthetic dark-blood T2IR-LGE, both the signals of myocardium and blood pool were almost completely nulled. Thus, both scar-to-blood and scar-to-myocardium contrast were significantly improved (close to 1). However, the blood-to-myocardium contrast was significantly reduced (close to 0). Therefore, the synthetic dark-blood T2IR-LGE could only be used to highlight the scar but not for the depiction of cardiac structure.

Compared with previous studies of synthetic LGE,^25,26^ the proposed method in this study achieved 3D imaging with isotropic resolution, which is beneficial for whole-heart assessment, especially for small lesions (Fig. 4). In addition, only T1 and T2 were included in previous studies for the LGE signal simulation, whereas this study adopted the proposed 3D joint T1/T2 mapping sequence to calculate T1, T2 and proton density simultaneously. By adding proton density contrast in the signal model, this study produced synthetic LGE images with a clearer background air region. The proposed signal models in this study aim to mimic the actual acquisition process of LGE imaging. Different signal models with specifically manipulated image contrast have been proposed in recent studies.^25,26^ Although not validated here, the proposed 3D joint T1/T2 mapping is also compatible with other signal models using T1 and T2. It is worth noting that some deep-learning-based methods have also been proposed to generate synthetic LGE images, such as synthetic bright-blood LGE using dark-blood LGE.^45^ Although the trained deep-learning model could be applicable to existing data without acquiring additional images, training the model requires a relatively large data set, which is difficult to acquire, especially for 3D imaging. The proposed method is based on physical signal models and can be directly applied to the acquired 3D T1/T2 maps without training.

In the case of myocardial infarction, the proposed method can detect scar area according to the alterations of T1 and T2 values and the hyperintense signal in synthetic LGE images. In addition, preliminary results of a swine in Figure 5 indicate that the proposed method may also allow the detection of MVO, which usually occurs in the sub-acute phase of myocardial infarction (1 d to 2 wk).^46^ In the area of MVO, T2 value is generally shorter than that of scar due to intramyocardial hemorrhage.^47^ However, T2 mapping alone may not have sufficient sensitivity for reliable MVO and hemorrhage detection;^46^ post-contrast T1 mapping and LGE imaging usually have a better image contrast as they are related to the distribution of the contrast agent. Here, both post-contrast T1 and synthetic LGE acquired with the proposed sequence showed the MVO area with higher T1 values and hypointense signal on LGE, although the MVO size was slightly different from that in 2D LGE. This difference could potentially because of the later timing of the 3D acquisition compared with 2D and the corresponding kinetics of the contrast agent. The hypointense and hyperintense area in LGE images are caused primarily by regional differences in wash-in/washout time constants.^48^ Usually, contrast wash-in in MVO is slower than that in scar. Therefore, when imaging at a later time after contrast injection, the detected area of MVO may be reduced as contrast begins to wash into the region of MVO. However, investigation of contrast kinetics was out of the scope of this study, and further experiments may be required before applying the proposed methods for MVO detection, especially optimizing the imaging time after contrast.

### Limitations

This study has some limitations. Firstly, 2D LGE, 2D T1 mapping, and 3D joint T1/T2 mapping were acquired sequentially after injection of gadolinium contrast and may therefore introduce some bias in the comparison of techniques. As post-contrast T1 is sensitive to the time after contrast injection, random scan order is preferred in future studies. ECV measurements should also be investigated in the future as it is more robust to the imaging time than post-contrast T1, although additional acquisition of pre-contrast T1 and hematocrit is required. In addition, 2D T2 mapping was acquired before contrast and compared with the post-contrast 3D joint T1/T2 mapping. The performance of post-contrast T2 measurements may require further validations. Secondly, although isotropic resolution was achieved, the resolution was limited to 2 mm due to the relatively lower SNR at low field. Further investigations are required to improve the spatial resolution in the future. Lastly, the experimental cohort is limited to a small sample size of 6 swine with myocardial infarction in the left circumflex artery territory alone. Further assessment on patients with different presentations of focal and diffuse infarct scar are required. For example, patients with subendocardial or papillary muscle scar are warranted to demonstrate the advantages of synthetic dark-blood LGE. In addition, the added value of T1/T2 mapping for the detection of diffuse disease requires to be explored in future works. All the data (n = 10) acquired at either 1-week (n = 6) or 5-week (n = 4) post-MI were combined for analysis due to the small sample size. Further experiments are warranted to investigate the differences in T1/T2 values between 1 week and 5 weeks.

## CONCLUSIONS

A 3D free-breathing joint T1/T2 mapping sequence was used to enable simplified and efficient simultaneous post-contrast parametric mapping and synthetic LGE imaging in a single scan at 0.55T. The findings were consistent with separately acquired 2D reference sequences. Results from swine experiments in this study are promising for comprehensive myocardial tissue characterization, and further validation in patients is warranted.

## Supplementary Material

Table S1

Supplemental Digital Content is available for this article. Direct URL citations are provided in the HTML and PDF versions of this article on the journal's website, www.investigativeradiology.com.

## Figures and Tables

**FIGURE 1.**
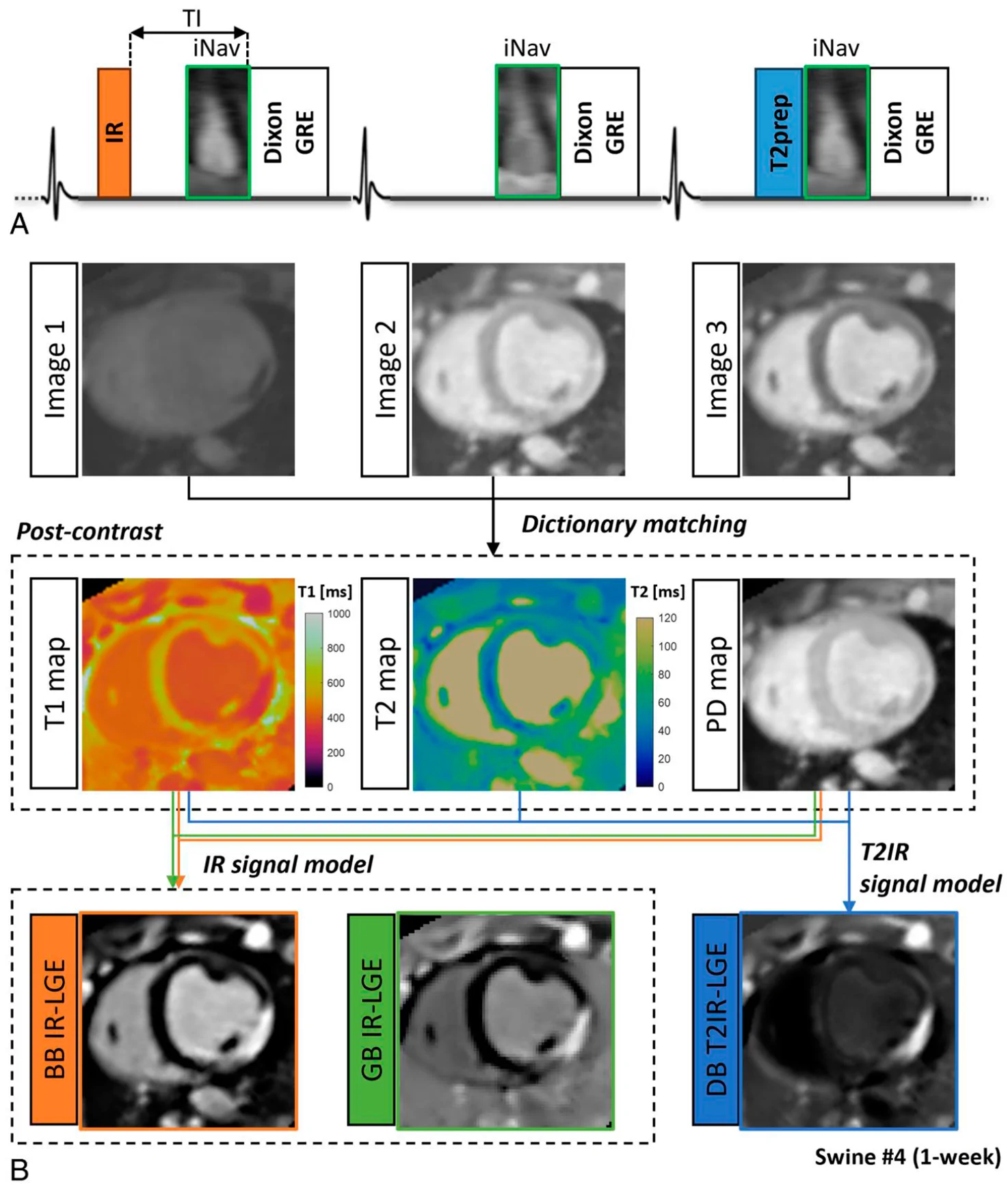
Framework of the proposed method for 3D joint T1/T2 mapping and synthetic multi-contrast LGE imaging. A, Pulse sequence diagram. Three electrocardiogram-triggered volumes are acquired with inversion recovery (IR) preparation (TI = 150 ms), no preparation, and T2 preparation (50 ms), respectively. Two-dimensional image navigators (iNAVs) are performed for respiratory motion compensation with 100% respiratory scan efficiency. Gradient echo (GRE) with 2-point bipolar Dixon acquisition and 4-fold undersampled variable-density Cartesian trajectory with spiral-like profile order is adopted. B, Calculation pipeline of multi-parametric and multi-contrast images. Intrinsically co-registered 3D T1, T2, and proton density (PD) maps are jointly calculated voxel-by-voxel from the 3 water volumes using a dictionary-matching method. An IR signal model is proposed to generate bright-blood (BB) and gray-blood (GB) IR-LGE using T1 and PD, whereas a joint T2 preparation IR (T2IR) signal model is proposed to generate dark-blood (DB) T2IR-LGE using T1, T2, and PD.

**FIGURE 2.**
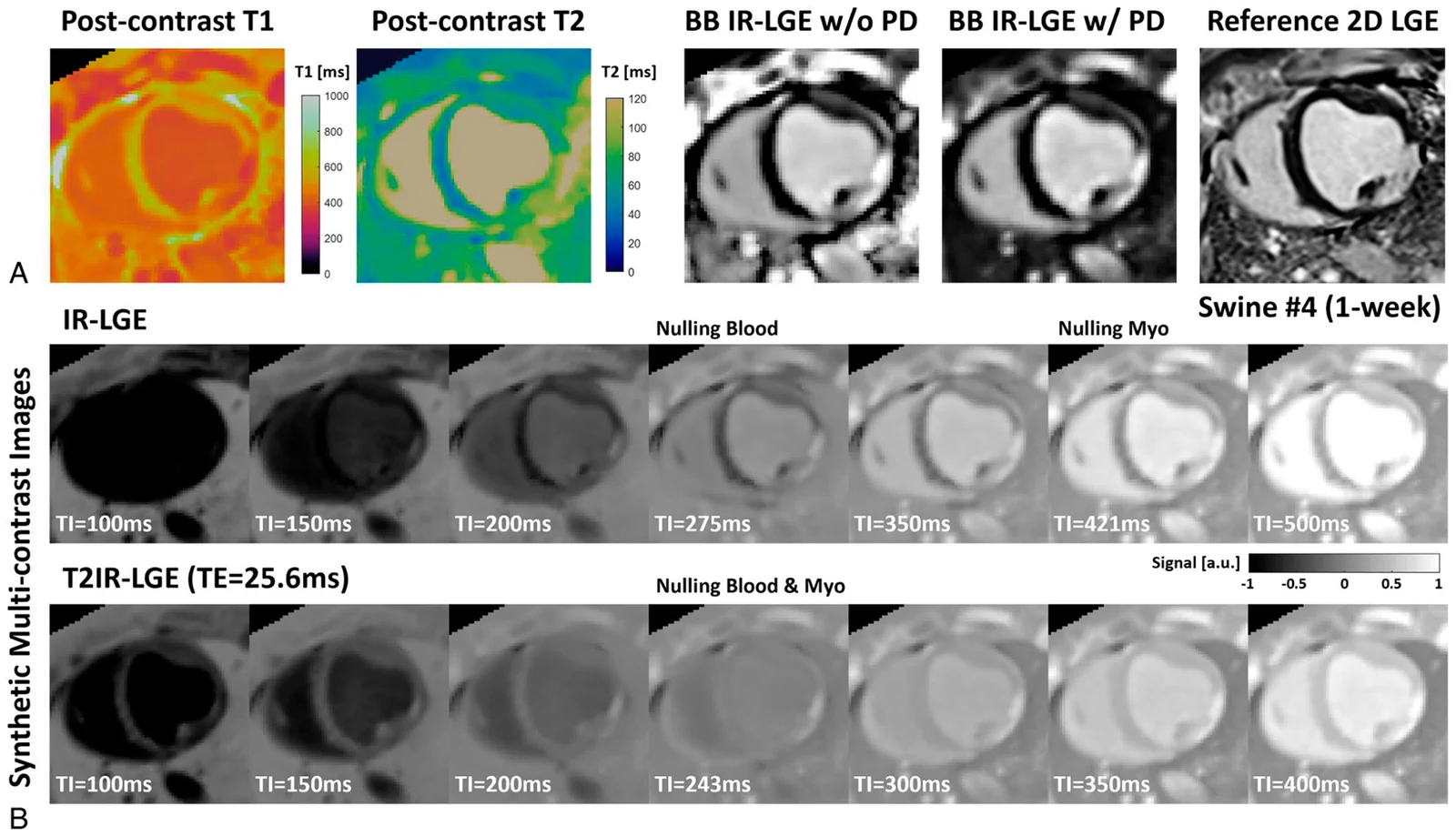
Representative T1 and T2 maps and synthetic multi-contrast LGE images of a swine produced with the proposed method. A, T1 and T2 maps and the corresponding synthetic bright-blood (BB) IR-LGE images calculated with and without proton density (PD) map in comparison with reference 2D LGE. Synthetic LGE with PD presents a cleaner background air region than that without PD and has results comparable with reference 2D LGE. B, Synthetic multi-contrast images with different signal model and different parameters. For IR-LGE, the blood signal is nulled with TI of 275ms, whereas the myocardium signal is nulled with TI of 421ms. IR-LGE images with inversion recovery time (TI) ranging from 100ms to 500ms are shown. For T2IR-LGE, the signal of blood and myocardium are simultaneously nulled with T2 preparation duration (TE) of 25.6ms and TI of 243ms. T2IR-LGE images with TE of 25.6ms and TI ranging from 100ms to 400ms are shown.

**FIGURE 3.**
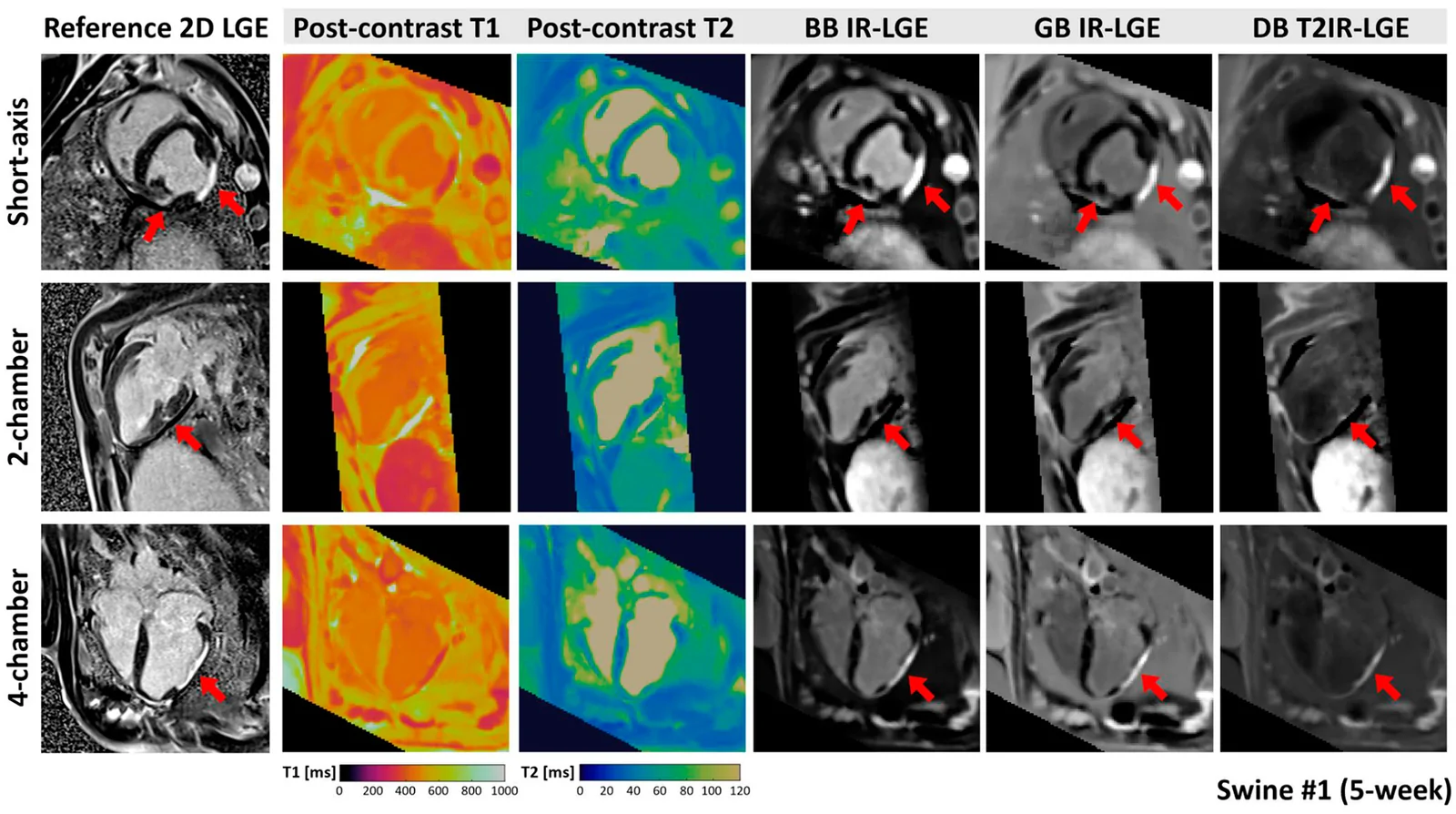
Representative co-registered T1/T2 maps and synthetic LGE images from another swine acquired with the proposed method in comparison with reference 2D LGE. 3D imaging with isotropic resolution enables reformatting in different orientations matching 2D LGE, including short-axis, 4-chamber, and 2-chamber views. Myocardial infarction scar is detected in 2D LGE as indicated by the hyperintense areas (red arrows). Correspondingly, 3D T1 and T2 maps show decreased T1 and increased T2 values in the scar areas, respectively. In addition, all synthetic bright-blood (BB) and gray-blood (GB) IR-LGE and dark-blood (DB) T2IR-LGE have findings of scar area that is consistent with reference 2D LGE, as shown by the red arrows. The synthetic BB IR-LGE has good image quality and image contrast comparable with reference 2D LGE. The synthetic GB IR-LGE had improved tissue contrasts for the depiction of all tissues. The synthetic DB T2IR-LGE has a relatively weak contrast for the depiction of cardiac structure as both blood and myocardium are suppressed; however, it presents an excellent contrast to spot scar.

**FIGURE 4.**
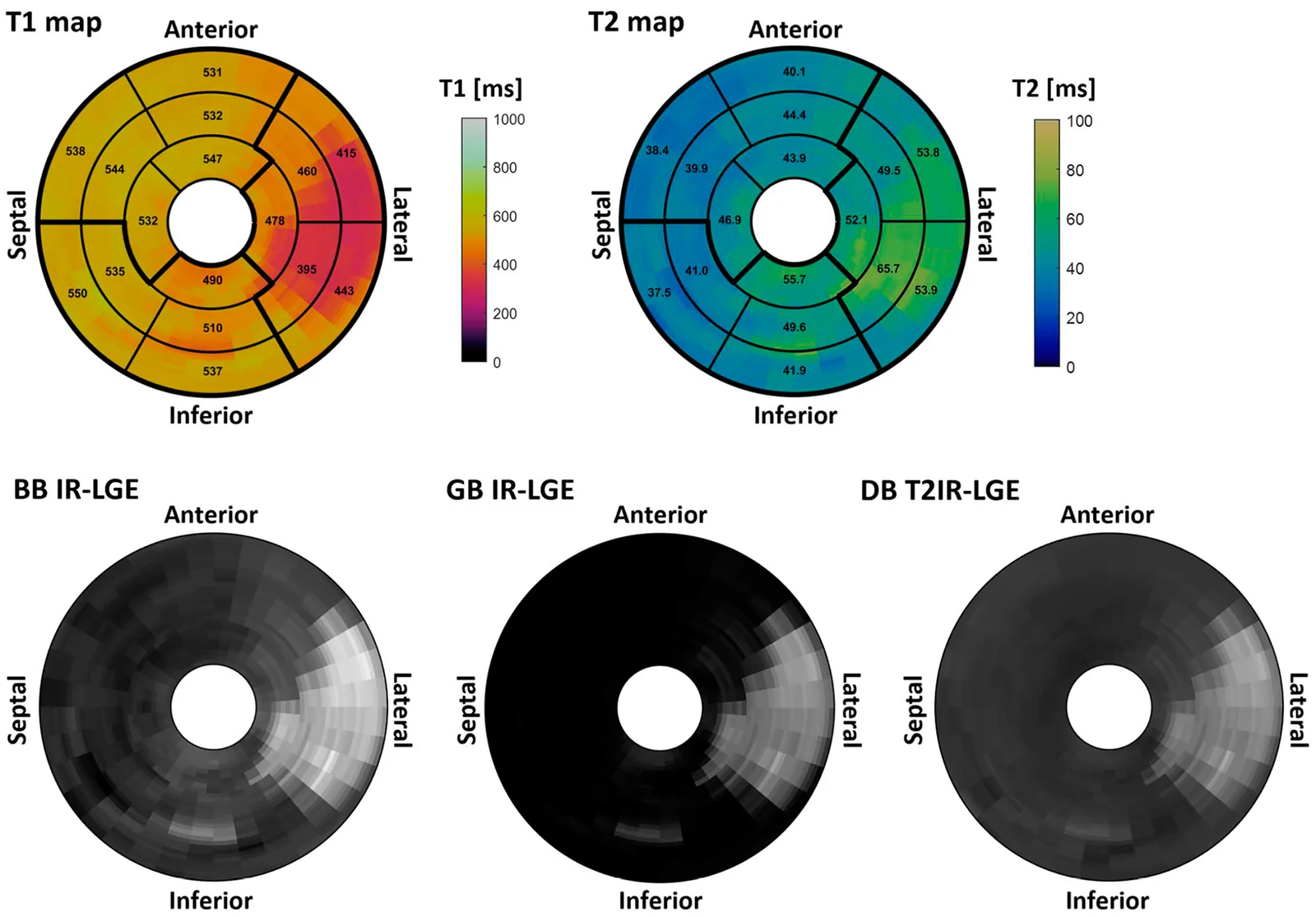
High-resolution bull’s-eye plots of T1/T2 maps and synthetic bright-blood (BB) and gray-blood (GB) IR-LGE and dark-blood (DB) T2IR-LGE images from the same swine shown in Figure 3. T1/T2 maps and synthetic LGE images show consistent findings in the segments with scar, demonstrating decreased T1 and increased T2 values and hyperintense, respectively. High-resolution plots allow an accurate depiction of the scar area across the whole heart.

**FIGURE 5.**
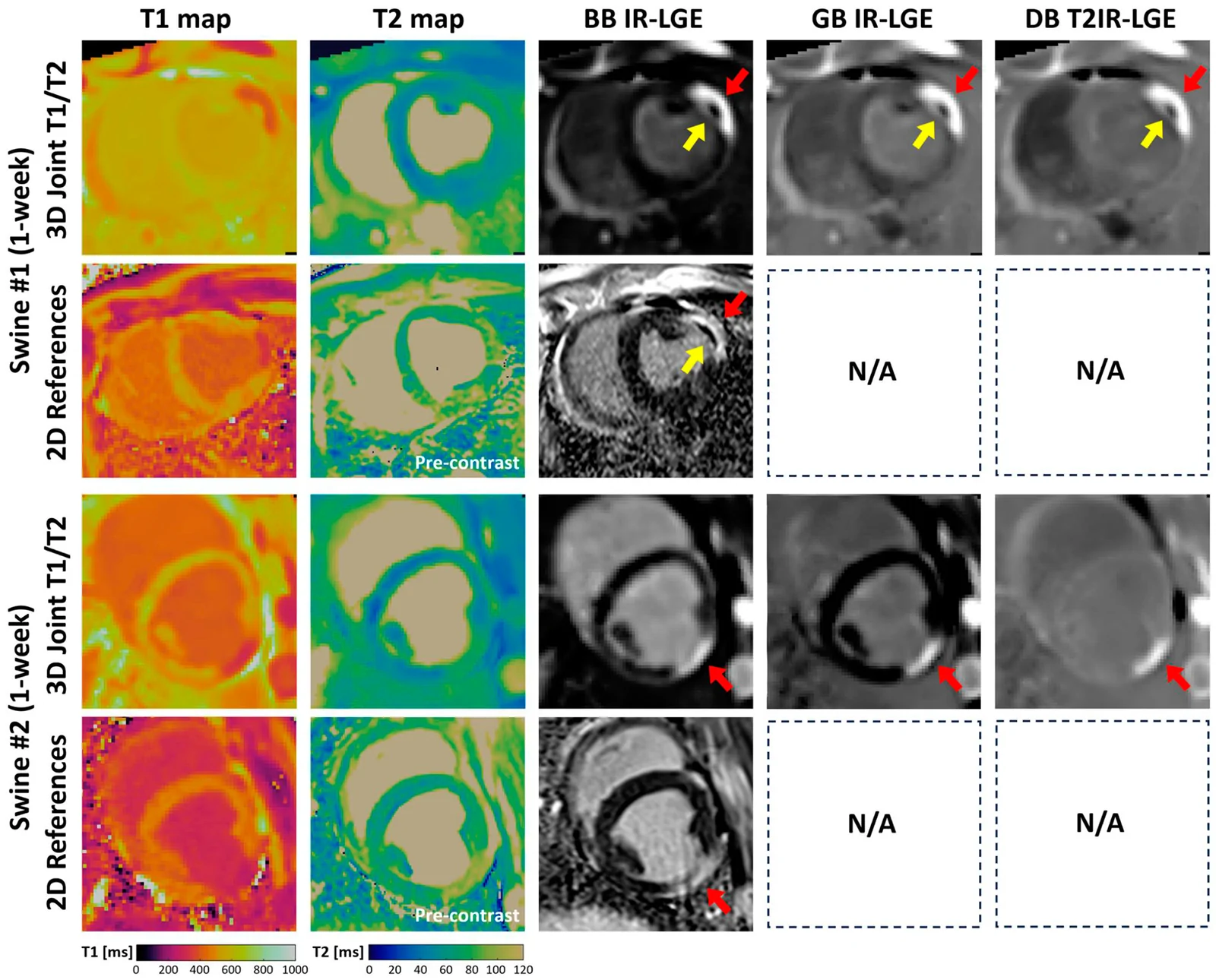
Short-axis T1/T2 maps and LGE images of additional 2 swine at 1-week post-myocardial infarction acquired with 2D and 3D sequences. For swine 1, 2D LGE shows a hyperintense area (red arrow) with a hypointense core (yellow arrow), indicating myocardial infarction with microvascular obstruction (MVO). All 3D synthetic bright-blood (BB) and gray-blood (GB) IR-LGE and dark-blood (DB) T2IR-LGE have similar findings of scar (red arrows) and MVO (yellow arrows). For swine 2, only myocardial infarction scar is presented as indicated by red arrows.

**FIGURE 6.**
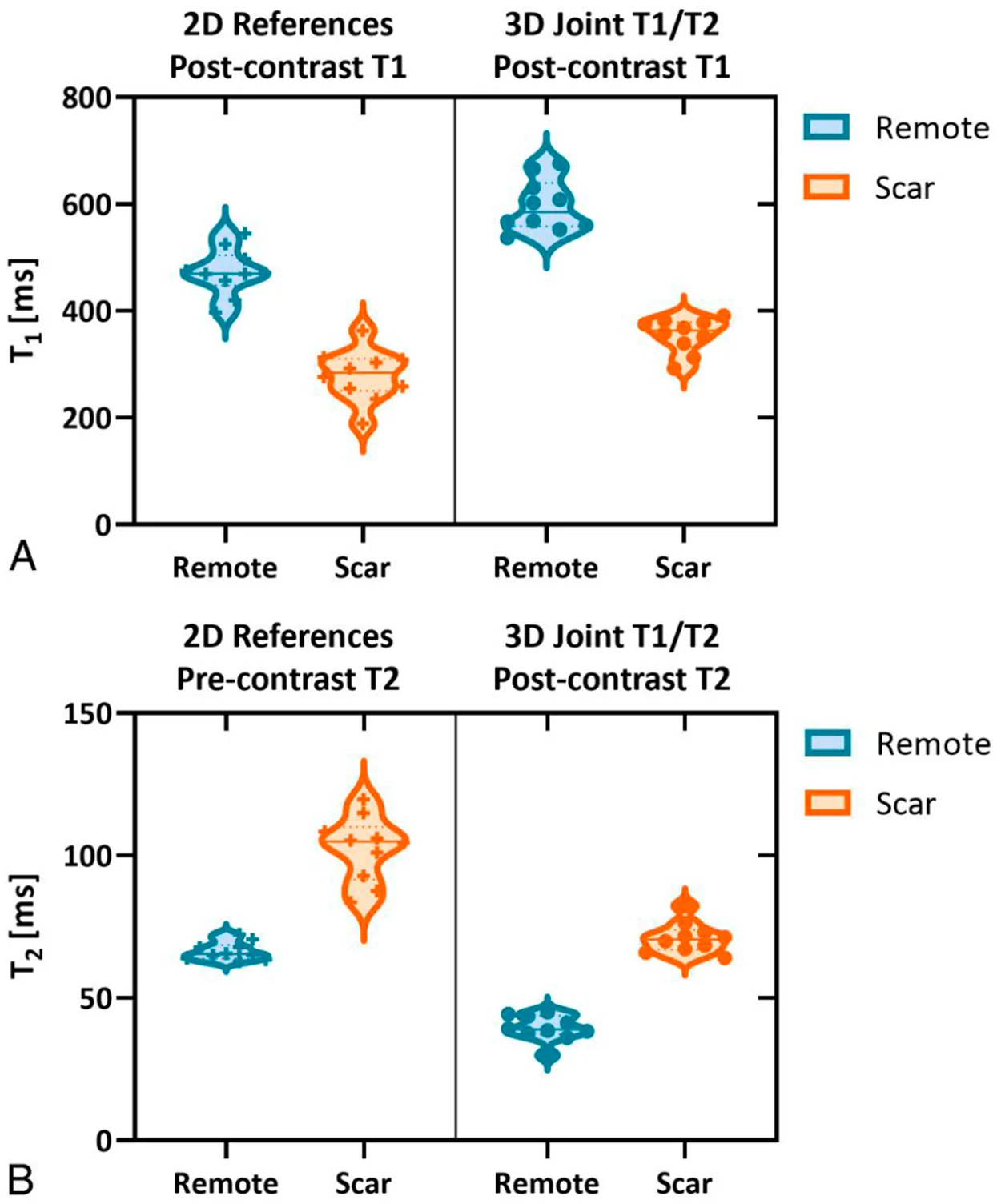
Violin plots comparing T1 (A) and T2 (B) of scar and remote myocardium measured by 2D and 3D sequences (n = 10).

**FIGURE 7.**
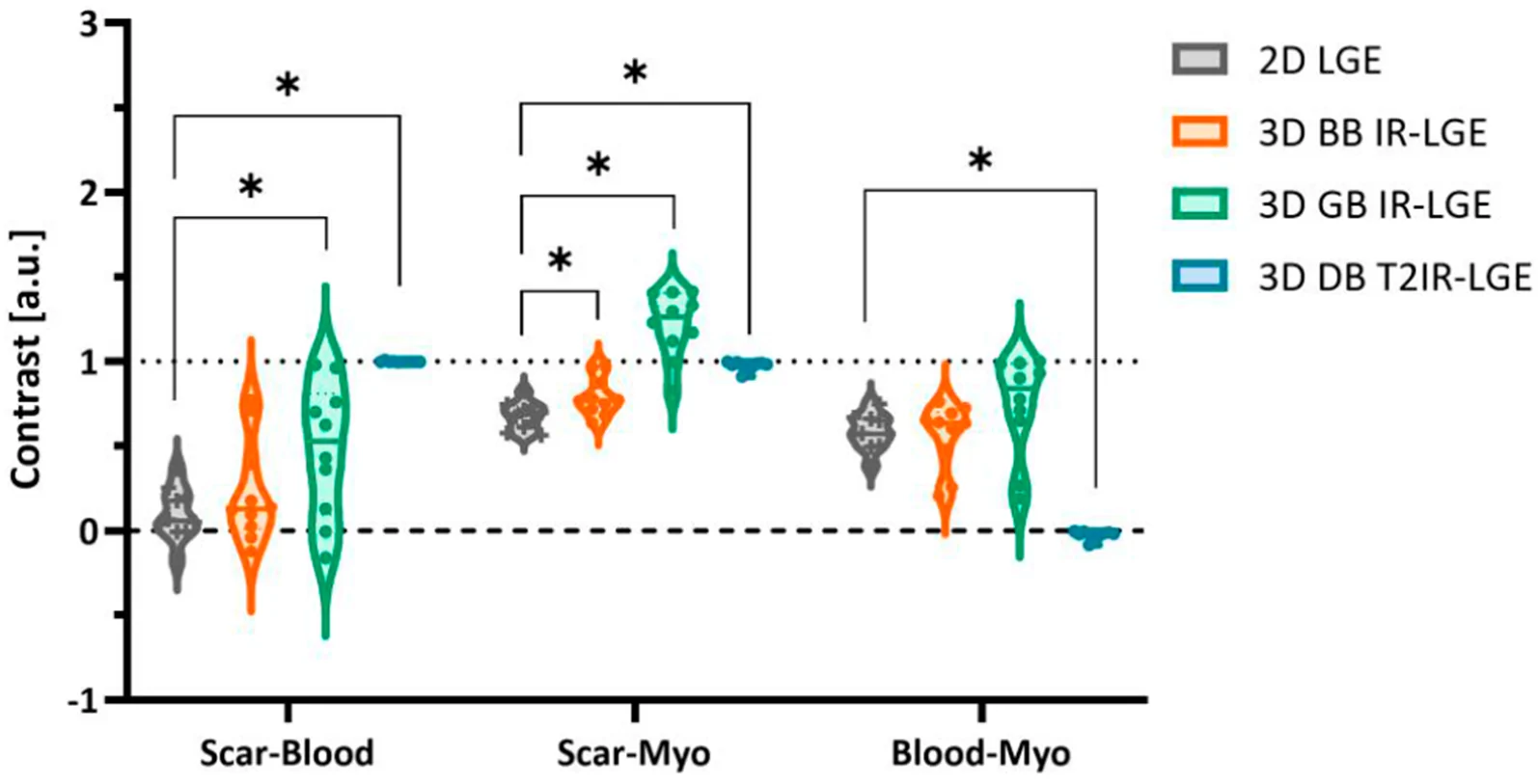
Violin plots comparing image contrasts of 2D LGE, 3D synthetic bright-blood (BB) and gray-blood (GB) IR-LGE and dark-blood (DB) T2IR-LGE (n = 10). * indicates significant differences according to paired 2-tailed Student *t* test.

**TABLE 1.** Comparison of T1 and T2 Values of Scar and Remote Myocardium Measured by 2D and 3D Sequences

	Blood	Remote Myocardium	Scar	*P* (Remote vs. Scar)
Post-contrast 2D T_1_	320 ± 71	472 ± 44	279 ± 48	< 0.01
Post-contrast 3D T_1_	423 ± 96	597 ± 48	355 ± 32	< 0.01
Pre-contrast 2D T_2_	279.0 ± 71.8	66.4 ± 3.1	102.4 ± 11.5	< 0.01
Post-contrast 3D T_2_	148.4 ± 18.2	39.4 ± 4.5	71.0 ± 5.3	< 0.01

N = 10 swine data sets acquired at either 1-week (n = 6) or 5-week (n = 4) post-myocardium infarction were included. *P*-values were calculated with a paired Student *t* test between remote myocardium and scar.
