# Supplementary material for: 3D Simultaneous Post-contrast T1/T2 Mapping and Synthetic Multi-Contrast Late Gadolinium Enhancement at 0.55T: Validation in Porcine Myocardial Infarction Model
Source: Invest Radiol. 2026 Feb 16;61(10):706–15. doi: 10.1097/RLI.0000000000001277 (PMC13255184; doi:10.1097/RLI.0000000000001277)
Supplement: Supplementary file 1 [file rli-61-706-s001.docx]

**Table S1.** Sequence parameters in swine experiments.

| Characteristic | 2D PSIR LGE | 2D T_1_ | 2D T_2_ | 3D joint T_1_/T_2_ |
| --- | --- | --- | --- | --- |
| Field of view | 262×350 mm^2^ | 240×320 mm^2^ | 240×320 mm^2^ | 320×320×~100 mm^3^ |
| Acquisition resolution | 2.1×1.5 mm^2^ | 2.3×1.7 mm^2^ | 1.7×1.7 mm^2^ | 2 mm^3^ |
| Reconstruction resolution | 0.7×0.7 mm^2^ | 1.7×1.7 mm^2^ | 0.8×0.8 mm^2^ | 2 mm^3^ |
| Slice thickness (mm) | 8 | 8 | 10 | NA |
| Readout | bSSFP | bSSFP | bSSFP | 2-point Dixon GRE |
| Acceleration | None | 2×GRAPPA | 2×GRAPPA | 4×VD-CASPR |
| Flip angle (°) | 80 | 35 | 70 | 8 |
| TR/TE (msec) | 5.8/2.28 | 4.83/1.99 | 4.45/1.71 | 9.72/2.65,6.5 |
| Bandwidth (Hz/pixel) | 302 | 543 | 554 | 401 |
| Segments | 25 | 54 | 40 | 14~16 |
| Preparation pulses | IR | IR | T_2_-prep | IR/none/T_2_-prep |
| No. of preps | 1 | 11 | 6 | 3 |
| Respiratory | Breath-hold | Breath-hold | Breath-hold | iNAV |

PSIR, phase-sensitive inversion recovery; LGE, late gadolinium enhancement; bSSFP, balanced steady-state free precession; GRE, gradient echo; GRAPPA, generalized autocalibrating partially parallel acquisitions; IR, inversion recovery; iNAV, image navigator.

**Table S2.** Total scan time and imaging time after contrast administration of different 2D and 3D sequences in this study.

|  | **Scan time (min)** | **Time after contrast (min)** |
| --- | --- | --- |
| Pre-contrast |  |  |
| 2D T2 | 6.4 ± 1.0 | N/A |
| Post-contrast |  |  |
| 2D LGE | 8.6 ± 1.8 | 13.8 ± 1.9 |
| 2D T1 | 7.1 ± 1.4 | 32.2 ± 5.2 |
| 3D joint T1/T2 | 5.0 ± 1.0 | 38.4 ± 5.6 |
| 2D in total | 22.2 ± 3.4 | N/A |

LGE, late gadolinium enhancement

**Figure S1.** Bland-Altman analysis comparing mean T1 (A, B) and T2 (C, D) of remote myocardium and scar measured by 3D joint T1/T2 mapping and conventional 2D sequences.
